# Use of regional citrate anticoagulation with medium cut-off membrane: pilot report

**DOI:** 10.1186/s12882-022-02960-y

**Published:** 2022-10-27

**Authors:** Marija Malgaj Vrečko, Jernej Pajek, Jadranka Buturović-Ponikvar

**Affiliations:** 1 Department of Nephrology, University Medical Centre Ljubljana, Ljubljana, Slovenia; 2 Faculty of Medicine, University of Ljubljana, Ljubljana, Slovenia; 3 Department of Nephrology, University Medical Center Ljubljana, Zaloška cesta 7, 1000 Ljubljana, Slovenia

**Keywords:** Expanded hemodialysis, Anticoagulation, Citrate, Calcium, Magnesium

## Abstract

**Background:**

Regional citrate anticoagulation during hemodialysis provides an immediate and complete anticoagulant effect, which is limited to the extracorporeal circuit. Citrate has become the standard anticoagulant in acute renal replacement therapy and is widely used in various intermittent hemodialysis modalities, especially for patients with contraindications for heparin. With the increased adoption of medium cut-off membranes, experience with regional citrate anticoagulation is needed. To our knowledge, this is the first report to assess the feasibility of regional citrate anticoagulation in expanded hemodialysis.

**Methods:**

We prospectively analyzed 5 expanded hemodialysis procedures in 5 patients in which a medium cut-off membrane (Theranova®) was used. We followed our standard citrate protocol developed and tested for high-flux membrane. Anticoagulation was performed with a continuous infusion of 8% trisodium citrate into the arterial line and supplementation of 1 M calcium chloride into the venous line. We monitored ionized calcium and magnesium, sodium and blood gas analysis. Anticoagulation effectiveness was assessed by post-filter ionized calcium and by visual inspection of the anticoagulation in the circuit.

**Results:**

There were no prematurely terminated procedures due to anticoagulation-related complications. With a blood flow of 250 mL/min and a dialysate flow of 500 mL/min, we were able to maintain serum ionized calcium in the range of 0.89–1.29 mmol/L and serum sodium in the range of 136–144 mmol/L. The mean pre- and post-dialysis arterial circuit pH was 7.42 (± 0.04) and 7.53 (± 0.23), respectively. The mean pre- and post-dialysis serum ionized magnesium was 0.54 (± 0.04) mmol/L and 0.43 (± 0.03) mmol/L, respectively (measurements were done on a point-of-care ionometer with a lower normal range for ionized magnesium).

**Conclusion:**

We have shown that our standard citrate protocol for high-flux hemodialysis membrane could be successfully adopted for use in expanded hemodialysis with a medium cut-off membrane. Overall, electrolyte and acid-base balances were relatively well-controlled and anticoagulation effectiveness was excellent.

**Trial registration:**

This is a pilot report with results taken from a larger ongoing trial (registered at ClinicalTrials.gov on October 25, 2019 under number NCT04139525) comparing citrate and heparin anticoagulation during expanded hemodialysis.

## Background

Regional citrate anticoagulation (RCA) during hemodialysis (HD) provides an excellent anticoagulant effect that is immediate, complete, and limited to the extracorporeal circuit. Citrate exerts its anticoagulant effect by chelation of calcium (an essential factor in the coagulation cascade) and consequently induces severe hypocalcemia in the dialysis circuit. Because the anticoagulant effect of citrate is limited to the extracorporeal circuit, RCA is primarily used in patients who have contraindications for heparin anticoagulation (e.g., a high risk of bleeding, heparin-induced thrombocytopenia). Inhibition of coagulation cascade is better in comparison to heparin anticoagulation. [1] Moreover, the depletion of calcium in the extracorporeal circuit reduces many of the calcium-dependent blood-surface interactions. Accordingly, citrate has important effects beyond anticoagulation: it is known to improve the biocompatibility of the HD procedure by reducing leukocyte [2], platelet [2] and complement activation [3]. In addition, complement activation is also inhibited through citrate’s chelation of magnesium: ionized magnesium (iMg) represents an essential cofactor in the complement cascade, in particular in the alternative complement pathway, which is activated upon contact of blood with biomaterials. [4]

Another benefit of citrate is its size: the molecular weight of trisodium citrate is 294 Da. It is thus easily removed during HD: its removal during high-flux HD was shown to be more than 80%. [5] Exogenous citrate is metabolized into bicarbonate in the liver, muscles and kidneys. The metabolism of citrate is intact in HD patients.

The limitations of RCA are the technical elaborateness of the procedure and metabolic complications, such as hypo- or hypercalcemia, hypernatremia, hypomagnesemia and metabolic alkalosis. However, the risk of these complications has been importantly reduced by the use of high-flux dialyzers with high citrate clearance and sodium and bicarbonate adjustments offered by advanced HD monitors. Also, bedside control of ionized calcium (iCa) and iMg with point-of-care (POC) ionometers, and hence the adjustment of calcium and citrate infusion rates, has improved the safety of RCA. With all this in mind, severe metabolic complications can be easily prevented in the majority of patients. [6]

Given all its benefits, citrate has already become the standard anticoagulant in acute kidney injury requiring continuous renal replacement therapy, not only for adults [7], but also for pediatric patients [8]. Furthermore, RCA is being increasingly used in various intermittent HD modalities, such as chronic high-flux [6, 9, 10] and low-flux HD [11], hemodiafiltration with a high cut-off (Theralite®) membrane [12], and single-needle HD [13].

Although there has been increased adoption of medium cut-off (MCO) membranes in recent years [14–16], we have found no reports in literature of RCA in expanded hemodialysis (HDx). The primary aim of this report was therefore to assess the feasibility of RCA in HDx.

## Methods

We prospectively analyzed 5 HDx procedures performed from September to November 2020 on 5 chronic HD patients (3 females and 2 males, average age 56 ± 9 years (range 42–66 years)). This is a pilot report on the effectiveness and safety of RCA with HDx modality with results taken from a larger ongoing trial comparing citrate and heparin anticoagulation during HDx (the trial was registered at ClinicalTrials.gov on October 25, 2019 under registration identification number NCT04139525). Research was conducted ethically in accordance with the World Medical Association Declaration of Helsinki and was approved by the Medical Ethics Committee of the Republic of Slovenia (approval number 0120 − 11/2019/3). Written informed consents were obtained from all patients for participation in the study and publication of this report. None of the patients had any medical contraindications or specific indications for RCA, such as known citrate intolerance or high risk of bleeding. The mean duration of HDx was 261 ± 20 min (Table 1). In all cases, an arteriovenous fistula was used as vascular access. We used a MCO polyarylethersulphone-polyvinylpirrolidone membrane: Theranova® 400 in 3 and Theranova® 500 in 2 cases.

Blood flow was set at 250 ml/min, and a calcium-free, magnesium 0.50 mmol/l dialysate was used at a flow of 500 ml/min. Ultrafiltration rate was set according to the patient’s therapeutic needs. We used our standard citrate protocol developed and tested for high-flux HD membrane. RCA was performed with a continuous infusion of 8% trisodium citrate (prepared by our hospital pharmacy) into the arterial line at 150 ml/h and 1 M calcium chloride into the venous line, starting at 13 ml/h. The calcium infusion rate was adjusted after each hourly iCa measurement by 1–2 ml/h (in accordance with our clinical experience and without a pre-specified scheme) to achieve the iCa target range of 0.95–1.2 mmol/l. Dialysate sodium was set at 138 mmol/L and bicarbonate at 28 mmol/L (variations were allowed according to a patient’s acid-base and electrolyte status). We measured iCa and sodium at the start of HD, after every full hour of HD, and at the end of HD. A blood gas analysis and measurements of iMg were done before and after HD. Blood for these measurements was taken from the circuit pre-filter arterial line. Blood gas analysis and sodium measurements were done on an i-STAT® 1 analyzer (Abbott Point of Care Inc., Abbott Park, United States of America), while iMg was measured on a Stat Profile® Prime ES analyzer (Nova Biomedical, Waltham, United States of America). For measurements of iCa we used either one of the mentioned POC ionometers. To emphasize, both POC ionometers were located at the dialysis center, allowing us to implement bedside control of electrolytes and acid-base status.

Anticoagulation was monitored by iCa measured after the dialyzer (post-filter iCa) after 30 min of HD on one of the mentioned ionometers; the target range was 0.25–0.35 mmol/l. Additionally, blood clotting time was measured by slide method after 2 h of HD, samples were taken from the venous line. The target range was 15–20 min. Visual assessment of the anticoagulation in the circuit was performed after HD by an experienced dialysis nurse, using a semi-quantitative score, at 3 points: the dialyzer, the arterial bubble trap, and the venous bubble trap. For arterial and venous bubble traps, scoring was graded as 5 = no visible clotting, 4 = fibrin ring, 3 = small clot (< 2 mL), 2 = big clot (> 2 mL), and 1 = total occlusion. For the dialyzer, the scoring was 5 = < 20 fibers clotted, 4 = 21–50 fibers clotted, 3 = 51–100 fibers clotted, 2 = > 100 fibers clotted, and 1 = > 20% of fibers clotted. Anticoagulation was considered optimal if the score at each point was 4 or higher. This kind of visual assessment was already used in our center’s previous trials [17, 18].

## Results

There were no prematurely terminated procedures due to RCA-related complications. Overall, serum iCa levels were relatively stable during the procedures, and other electrolyte and acid-base balances were well-controlled (Table 1). We were able to maintain serum ionized calcium in the range of 0.89–1.29 mmol/L, and serum sodium in the range of 136–144 mmol/L. Mean pre- and post-dialysis arterial circuit pH was 7.42 (± 0.04) and 7.53 (± 0.23), respectively. Mean pre- and post-dialysis serum ionized magnesium was 0.54 (± 0.04) mmol/L and 0.43 (± 0.03) mmol/L, respectively. There was only one case of hypocalcemia (< 0.90 mmol/l); the patient was asymptomatic. There were no cases of severe hypocalcemia (iCa < 0.80 mmol/l) or hypercalcemia (iCa > 1.30 mmol/l). Three cases of mild post-dialysis hypomagnesemia (iMg < 0.45 mmol/l) occurred (Table 1). No cases of hypernatremia (sodium > 146 mmol/l) due to RCA were observed. We had two cases of mild combined respiratory/metabolic alkalosis, both of them asymptomatic.

**Table 1:** Summary of electrolyte and acid-base balance in expanded hemodialysis with regional citrate anticoagulation

Patient	Size of Theranova membrane	Duration of HDx *(min)*	Blood flow *(mL/min)*	Dialysate flow *(mL/min)*	Pre-procedure iCa (mmol/L)^*^	Post-procedure iCa (mmol/L)^*^	iCa range (mmol/L)*	Pre-procedure iMg (mmol/L)*	Post-procedure iMg (mmol/L)*	Serum sodium range (mmol/L)*	Pre-procedure arterial circuit pH	Post-procedure arterial circuit pH
1	400	240	250	500	1.29	1.13	1.03–1.29	0.55	0.42	138–143	7.38	7.43
2	400	270	250	500	1.08	0.89	0.89–1.08	0.60	0.42	137–141	7.45	7.93
3	400	240	250	500	1.22	0.96	0.96–1.22	0.53	0.41	138–144	7.41	7.54
4	500	285	250	500	1.01	1.24	0.90–1.24	0.48	0.47	136–143	7.48	7.34
5	500	270	250	500	1.14	1.07	1.06–1.14	0.55	0.45	141–143	7.39	7.41

^*^The normal ranges for measurements according to the POC ionometer manufacturer were: iCa 1.09–1.30 mmol/L, sodium 136–146 mmol/L and iMg 0.45–0.60 mmol/L

Visual assessment of the anticoagulation in the circuit was excellent at the arterial and venous bubble traps, as well as the dialyzer: in all procedures, the scoring grades for the arterial and bubble traps and the dialyzer were 5. This was in accordance with the measurements of post-filter iCa after 30 min of HD, which were in the range of 0.23 to 0.25 mmol/l. On the other hand, the blood clotting times measured by slide method after 2 h of HD varied strongly among patients (range: 13–23 min). No adjustment of citrate infusion rate was needed at all.

## Discussion

To our knowledge, this is the first report of the use of RCA in HDx. We specifically focused on the performance of our standard citrate protocol (developed and tested for high-flux HD membrane [17]) with MCO membrane.

In order to avoid high citrate infusion rate to the patient our standard citrate protocol includes the use of calcium-free dialysate and blood flow of 250 mL/min with a consequentially moderate citrate infusion rate. If necessary, higher blood flow rates with increased citrate infusion rate can be safely used, because of high citrate clearance through dialysis membrane. [5] If citrate load is a concern, it may be avoided by stopping citrate 15–30 min before ending HD (in an “anticoagulant-free” dialysis mode), to allow remaining citrate to be dialyzed completely. It is also possible to use calcium-containing dialysate. The use of calcium-containing dialysate, although usually less complex and safer in comparison to the use of calcium-free dialysate (no risk of profound hypocalcemia), [19] has a problem of insufficient anticoagulation in dialysis circuit (especially venous part) despite higher citrate dose, as shown in our previous study. [20] Insufficient anticoagulation in turn reduces HD efficiency and may further complicate the procedure. Additionally, the citrate load on the patient may be higher. Potential beneficial effect of citrate anticoagulation to biocompatibility [2–4] may be decreased. In light of all this, we strongly believe that the use of calcium-free dialysate is superior to the use of calcium-containing dialysate during high-flux HD and HDx with RCA.

Previous studies have shown that small solute clearance is as effective with the MCO membrane as with high-flux membrane. [21, 22] Since calcium, magnesium and citrate are all small molecules, we did not expect any significant differences in behaviour of these molecules during HDx in comparison to high-flux HD. Therefore, we expected that our standard citrate protocol for high-flux membrane could also be used with MCO membrane without modifications. Our results show that the adoption of a standard citrate protocol developed for high-flux membranes could indeed be done without modifications and RCA could be a safe anticoagulation option in HDx. All in all, electrolyte and acid-base balances were relatively well-controlled and anticoagulation was excellent. However, the behavior of calcium and magnesium during HDx with RCA needs further attention.

Although mild hypocalcemia was allowed during the procedure (the lower limit for iCa was set at 0.95 mmol/l) in order to achieve a more neutral calcium balance, we had no cases of severe or symptomatic hypocalcemia and only one case of hypocalcemia, in which the patient was asymptomatic. There were no cases of hypercalcemia. We opted for the approach of allowing mild hypocalcemia because of previous data, which showed that maintaining values of iCa in the strictly nominative normal range during high-flux HD with RCA resulted in a significantly more positive calcium mass balance as compared to mild hypocalcemia during the procedure. [17] Aiming to achieve a more neutral calcium balance is especially important when RCA is used for prolonged periods in selected chronic HD patients. As we have not yet found any studies investigating this issue specifically on MCO membranes so far, our approach allowing mild hypocalcemia in patients undergoing HDx with RCA needs to be further validated.

In comparison with calcium balance, magnesium balance during intermittent HD has been studied in less detail: there is some data on the effect of RCA on serum magnesium levels in intermittent HD [12, 21], but the topic has received scant attention. [23] It is important to emphasize that hypomagnesemia is not to be ignored, since some cohort studies have reported that lower serum magnesium levels are associated with an increased risk of all-cause and cardiovascular mortality among HD patients. [24] Magnesium’s behavior during RCA is complex. As a divalent cation, magnesium is bound by citrate and its clearance is therefore increased in the form of citrate-magnesium complexes. However, the decrease in iMg could increase magnesium flux from the dialysate. [17] The results of patients on high-flux HD show that RCA may decrease serum magnesium levels, possibly due to a negative magnesium balance. [10, 17] Our findings show that a decrease in serum magnesium levels might occur in HDx as well: we observed three cases of mild, post-dialysis asymptomatic hypomagnesemia, which did not require magnesium substitution. It is expected, as citrate is metabolized to bicarbonate, that magnesium (as well as calcium) would be released after HD. To underline, the effect of RCA on serum magnesium levels needs to be further validated. Specifically, more attention needs to be given to the magnesium concentration in dialysate, or even additional magnesium supplementation. Calcium-free dialysate, which was used in our study and is also used for RCA in the majority of protocols, has a magnesium content of 0.5 mmol/l. Since citrate binds to both calcium and magnesium, an increase in dialysate magnesium concentration may require a higher citrate dose, so this is a complex issue.

Previous study, which was done in our center has shown that hypocalcemia (< 0.9 mmol/L) during high-flux HD with RCA is rare. [9] Although the risk of hypocalcemia according to the results presented here appears to be higher during HDx, it is impossible to justly compare these results as our number of patients was quite low. Judging from our personal observations with MCO membranes, it seems that the risk of hypocalcemia and hypomagnesemia with RCA is similar in high-flux HD and HDx, which is in accordance with comparable clearance of small solutes during both types of dialysis. [21, 22]

According to the visual assessment of anticoagulation in the circuit and the measurements of post-filter iCa, anticoagulation was excellent. Since the blood clotting times measured by slide method varied strongly among patients, the measurement of iCa in the circuit proved to be a more reliable indicator of anticoagulation. All in all, it seems that our protocol is optimal in regard to anticoagulation efficiency.

## Conclusion

In conclusion, we have shown that RCA during HDx is feasible and could be a safe anticoagulant option especially for selected patients with contraindications for heparin anticoagulation (e.g., high risk of bleeding and heparin-induced thrombocytopenia). The adoption of a standard citrate protocol developed for high-flux membranes could be done without modifications. Further investigation is necessary to improve the protocols for RCA in HDx, focusing on the prevention of metabolic complications.

## Data Availability

The datasets used and analyzed during the current study are available from the corresponding author on reasonable request.

## List of abbreviations

HD: hemodialysis.
HDx: expanded hemodialysis.
iCa: ionized calcium.
iMg: ionized magnesium.
MCO: medium cut-off.
POC: point-of-care.
RCA: regional citrate anticoagulation.
